# Cigarette smoke-induced disordered microbiota aggravates the severity of influenza A virus infection

**DOI:** 10.1128/msystems.00790-24

**Published:** 2024-11-20

**Authors:** Tsering Wüthrich, Simone de Brot, Veronica Richina, Nadja Mostacci, Zora Baumann, Nathan G. F. Leborgne, Aurélie Godel, Marco P. Alves, Mohamed Bentires-Alj, Charaf Benarafa, Markus Hilty

**Affiliations:** 1 Institute for Infectious Diseases, University of Bern, Bern, Switzerland; 2 Graduate School of Cellular and Biomedical Sciences, University of Bern, Bern, Switzerland; 3 COMPATH, Institute of Animal Pathology, University of Bern, Bern, Switzerland; 4 Department of Biomedicine, Department of Surgery, University Hospital Basel, University of Basel, Basel, Switzerland; 5 Institute of Virology and Immunology, Mittelhäusern, Switzerland; 6 Department of Infectious Diseases and Pathobiology, University of Bern, Bern, Switzerland; 7 Multidisciplinary Center for Infectious Diseases, University of Bern, Bern, Switzerland; Zhejiang University College of Animal Sciences, HangZhou, Zhejiang, China

**Keywords:** cigarette smoke, microbiota, airways, feces, H1N1 infection, germ free mice

## Abstract

Cigarette smoke (CS) promotes the development of chronic pulmonary disease and has been associated with increased risk for influenza-related illness. Here, we directly addressed the impact of CS disordered microbiota on the severity of influenza A virus (IAV) infection. Specific and opportunistic pathogen-free (SOPF) C57BL/6J mice were exposed to CS or room air (RA) for 5.5 months. Each exposed mouse was then cohoused with a group of recipient germ-free (GF) mice for 1 month for microbial transfer. Colonized GF mice were then infected intranasally with IAV and disease development was monitored. Upper and lower airway and fecal microbiota were longitudinally investigated by 16S rRNA gene sequencing and bacterial cultures in donor and recipient mice. The bacterial family *Streptococcaceae* accounted for the largest difference between CS- and RA-exposed microbiota in the oropharynx. Analysis of the oropharynx and fecal microbiota indicated an efficient transfer to coprophagic recipient mice, which replicated the differences in microbiota composition observed in donor mice. Subsequent IAV infection revealed significantly higher weight loss for CS microbiota recipient mice at 8–10 days post infection (dpi) compared to control recipient mice. In addition, H1N1 infection inflicted substantial changes in the microbiota composition, especially at days 4 and 8 after infection. In conclusion, mice with a CS-associated microbiota suffer from higher disease severity upon IAV infection compared to mice colonized with a normal SOPF microbiota. Our data suggest that independently of CS exposure and concomitant structural lung damage, microbial distortion due to CS exposure may impact the severity of IAV disease course.

**IMPORTANCE:**

It has been reported that chronic exposure to CS is associated with a disordered microbiota composition. In this study, we colonized germ-free (GF) mice with the microbiota from SOPF mice which were chronically exposed to CS or RA. This allowed disentangling the effect of the disordered microbiota from the immune-modulating effects of actual CS exposure. We observed a successful transfer of the microbiotas after cohousing including specific microbiota differences induced by CS exposure in formerly GF mice, which were never exposed to CS. We then investigated the effects of IAV infection on the disease course and microbiotas of formerly GF mice. We found that mice with CS-associated microbiota reveal worse disease course compared to the control group. We hypothesize that CS-induced disordering of the microbiota may, indeed, impact the severity of influenza A disease.

## INTRODUCTION

Cigarette smoking is a major contributor in the development and severity of chronic pulmonary and cardiovascular diseases, cancer, and infections. A continuous population health survey from Australia identified independent factors associated with influenza-like illness including smoking, young age, and obesity (1). Similarly, questionnaires from a Hong Kong study investigating male college students revealed that clinical influenza incidence among those who smoked 21 or more cigarettes daily was 21% higher than that of non-smokers (2).

The identified associations between smoking and increased risk for influenza have been described to be of mechanical, structural, and immunological nature (3–5). Using a mouse model, we previously showed that CS exposure alters the relative composition of the oropharyngeal microbiota and reduces its diversity (6). Several studies identified distinctive host–microbiota interactions unveiling associations between the microbiota composition, airway inflammation, and disease exacerbation (7–10). However, it is currently unknown if the disordered microbiota itself contributes to the risk for severe influenza A virus (IAV) disease because its effect cannot be disentangled from those induced by CS.

The microbiota influences immune defense against respiratory tract IAV infection (7). Experiments in which the microbiota was regulated by using various antibiotic treatments showed the importance of commensal microbiota in immunity through the activation of inflammasomes (11). Similarly, mice treated with antibiotics exhibit impaired innate and adaptive antiviral immune responses and substantially delayed viral clearance after exposure to systemic IAV infection (8). However, these studies did not investigate the relevance of the local microbiota during chronic CS exposure. Bacterial taxa Alloprevotella, Prevotella, and Bacteroides from the nasal and laryngeal microbiome were found to be associated with increased host susceptibility to IAV infection, but this seemed to be independent of the smoking status of the study participants (9). In a household transmission study, high abundance of *Streptococcus* spp. and *Prevotella salivae* in upper airway swabs were also associated with higher susceptibility to IAV infection (12). Furthermore, patients and ferrets infected with IAV exhibit large changes in bacterial community composition over time and between individuals. Interestingly, the unhealthy ecostate of infected individuals progressed toward the healthy ecostate, coinciding with viral clearance and recovery over the course of the experiment (10).

To disentangle the effect of the disordered microbiota from the immune modulating effects of actual CS exposure, we colonized germ-free (GF) mice with the microbiota of SOPF mice which were chronically exposed to CS or to room air (RA). After successful microbiota transfer, the recipient mice were inoculated with H1N1 IAV to reveal the effects of dysbiosis induced by CS on virus clearance and lung inflammation.

## RESULTS

### Development of disordered microbiota after chronic CS exposure

**Fig 1:**
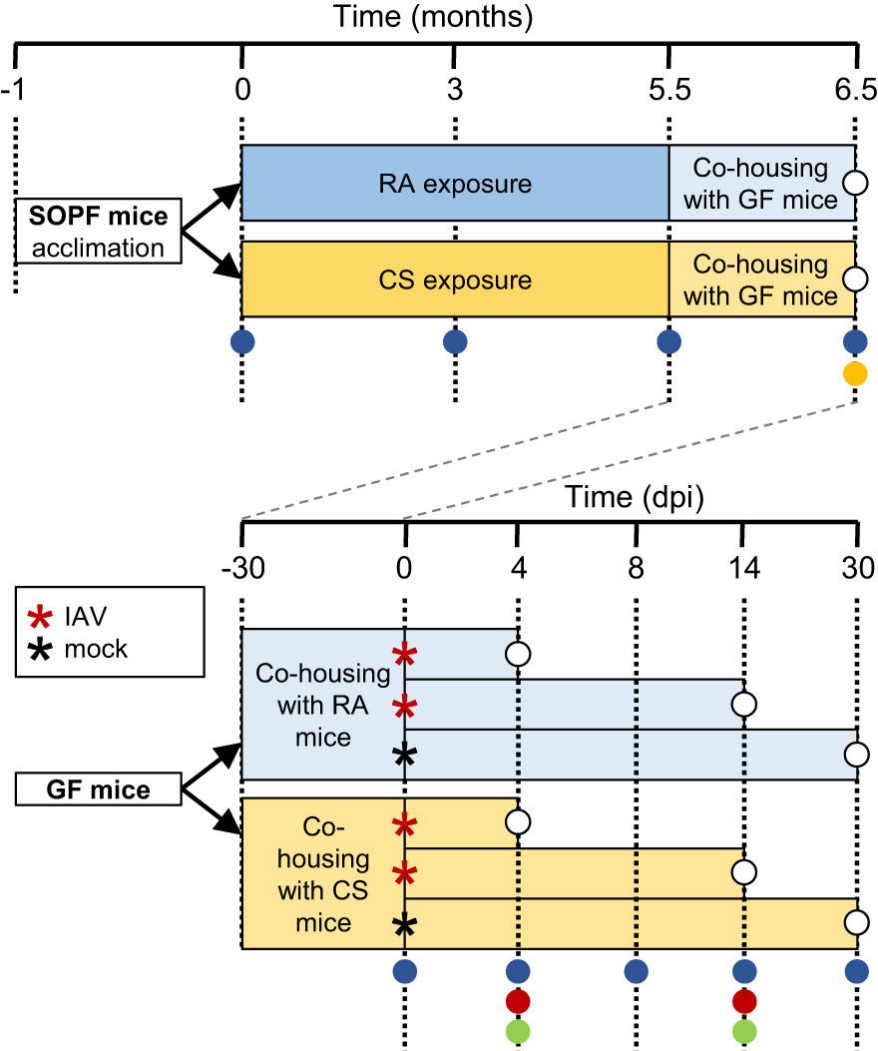
Experimental design. (**A**) Time line of exposure and sampling of microbiota donor mice. Four-week-old C57BL/6JRj SOPF female mice were randomized in two groups at delivery (*n* = 8/group) and acclimatized for 1 month before the start of exposure to cigarette smoke (CS; orange) or to room air (RA; blue) for 5.5 months. Mice in the CS group were exposed to CS generated by a Teague TE-10 smoking machine for two 90 min periods/day, 5 days/week. At the end of the exposure (5.5 months), each RA- and CS-exposed mouse was housed in a cage with 3 or 4 C57BL/6J germ-free mice, and the mice were left undisturbed for 1 month. SOPF donor mice were sampled at 0, 3, 5.5, and 6.5 months from the start of exposure. (**B**) Time line of microbiota transfer and IAV infection of GF mice. Upon delivery, 6–12-week-old C57BL/6J GF mice were immediately divided into individually ventilated cages (*n* = 3 or 4 GF mice/cage) containing a single microbiota donor mouse previously exposed to RA or CS. After 1 month of undisturbed co-housing, subgroups of GF mice were inoculated intranasally with 100 PFU IAV (red asterisk) or mock-infected (black asterisk). (**A and B**) Samples collected are indicated at the corresponding time points: microbiota analysis of oropharyngeal swabs and fecal pellets (blue circle), amplicon sequence variant and bacterial cultures of oropharyngeal and fecal cultures (yellow circle), viral and bacterial load in lung homogenate (red circle), microbiota analysis and histopathology of the lungs (green circles).

**Fig 2:**
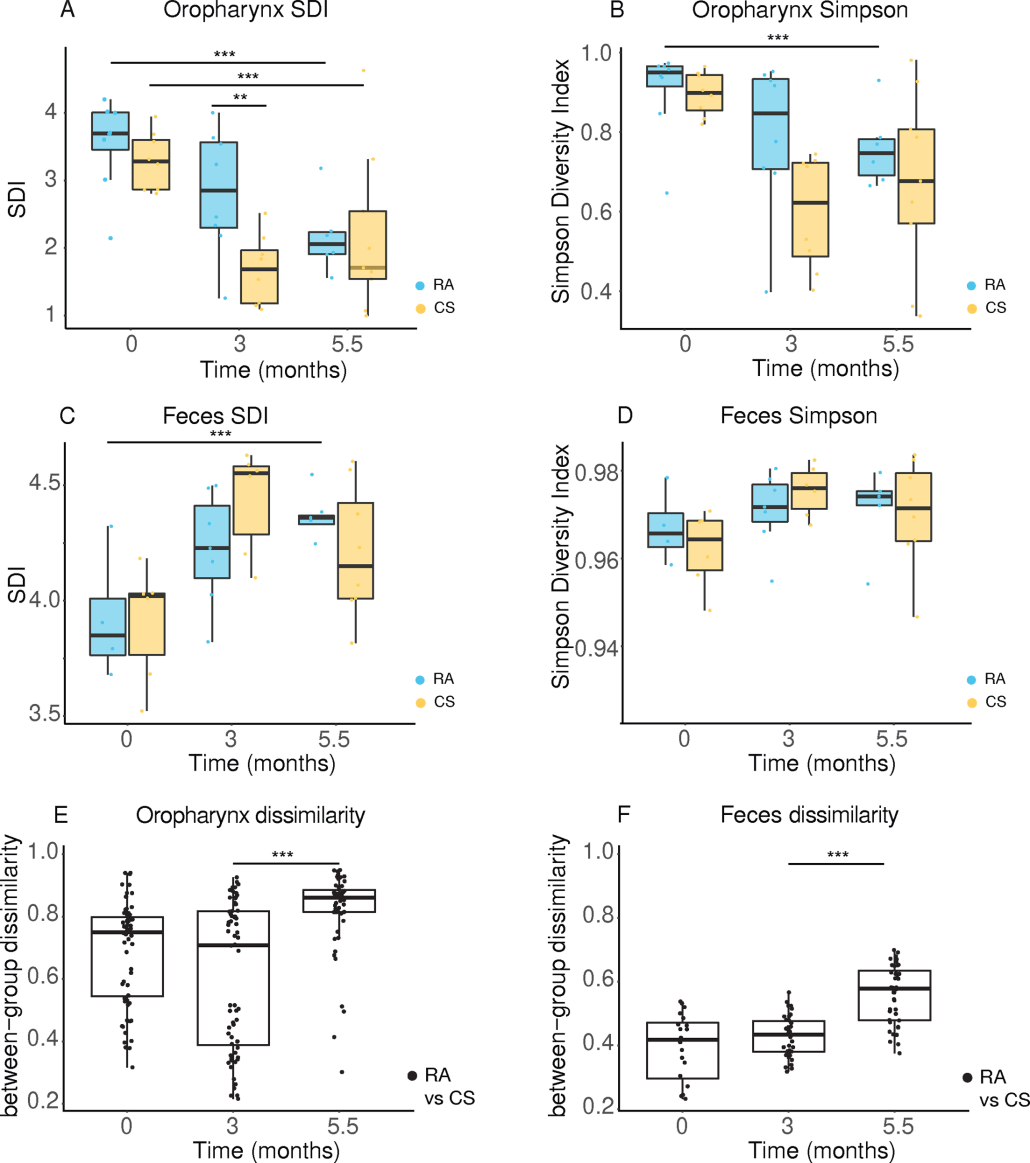
Chronic CS exposure induces changes in upper airway and gut microbiota diversity. (**A and B**) Alpha diversity values of the oropharynx microbiota shown as (**A**) Shannon diversity index (SDI) and (**B**) Simpson index of SOPF mice exposed to RA or CS. (**C and D**) Alpha diversity values for the fecal microbiota samples. (**E and F**) Beta diversity analysis calculated using Bray Curtis distance matrices for the oropharyngeal (**E**) and fecal (**F**) microbiota dissimilarity. Box-plots indicate median and interquartile range with mean indicated by + and with outliers shown. Data were analyzed by two-way ANOVA (**P < 0.001; ***P < 0.001).

To document the extent of microbiota disruption induced by chronic CS exposure, SOPF microbiota donor mice were exposed to standardized CS or to room air (RA) for 5.5 months. In both groups, the oropharyngeal and the fecal microbiota were sampled at the start of the study (month 0), and after CS or RA exposure for 3 and 5.5 months (Fig. 1). We found that the oropharynx microbiota alpha diversity, determined by Shannon and Simpson diversity indices, decreased over time for both groups (Fig. 2A and B). Notably, Shannon values decreased more rapidly in the CS-exposed group compared to the RA group as indicated for the 3 months’ time point (Fig. 2A). In contrast, SDI values in the feces increased over time in both groups, but there was no difference between the two groups at any time point (Fig. 2C and D). Overall, beta diversity and between-group dissimilarity values increased over time for oropharynx and feces, indicating the development of a divergence between CS-exposed microbiota compared to the RA control group (Fig. 2E and F).

**Fig 3:**
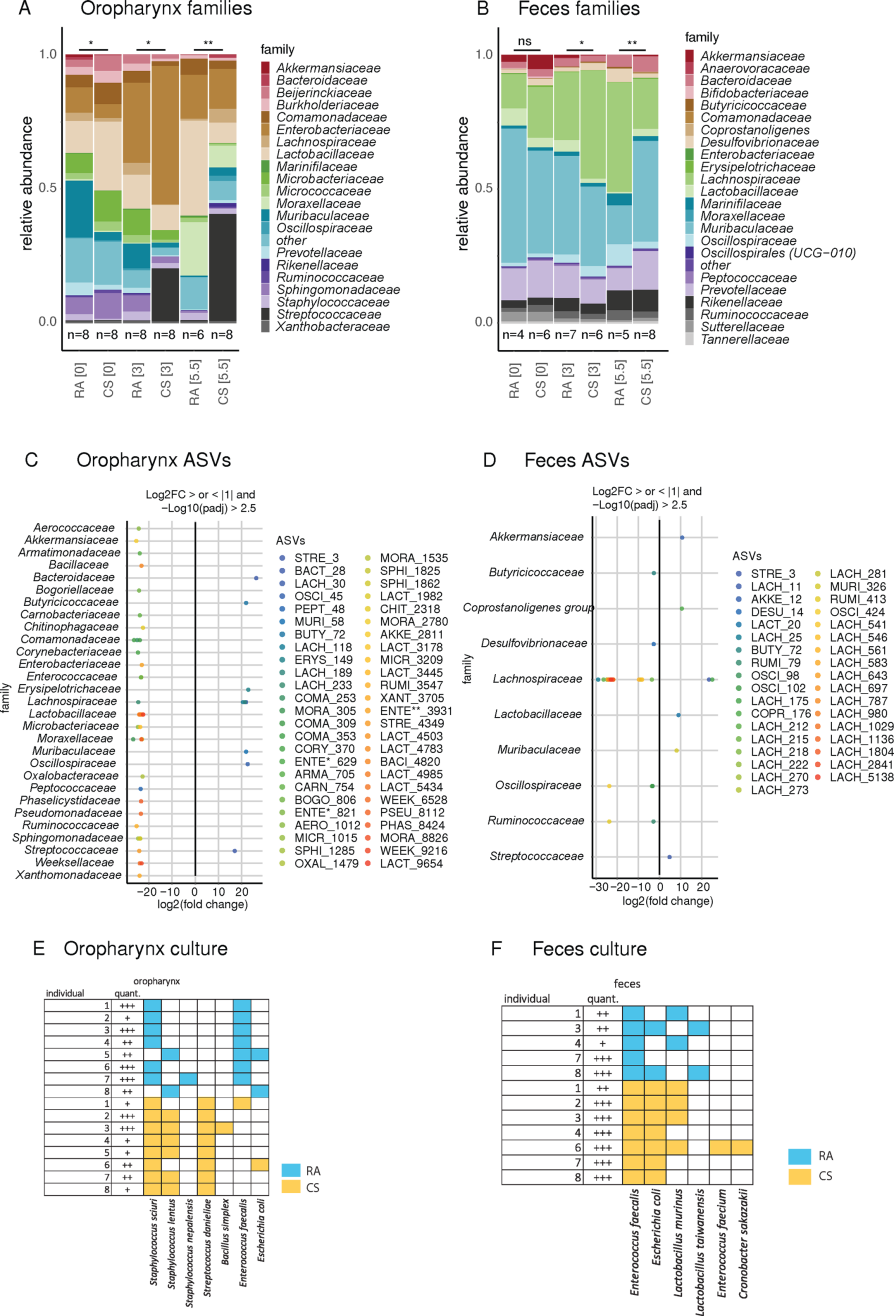
Chronic CS exposure induces quantitative and qualitative changes in oropharyngeal and fecal microbiota. (**A and B**) Relative abundances of the most prevalent bacterial families in (**A**) oropharyngeal swabs and (**B**) fecal pellets of SOPF mice exposed to RA or CS for 0, 3, 5.5 months. Data were analyzed using PERMANOVA tests (*P < 0.05; **P < 0.01). (**C and D**) Differential abundance of amplicon sequence variants (ASV) in (**C**) oropharyngeal swabs and (**D**) fecal pellets after 5.5-month exposure to RA or CS (*P*-adjusted < 0.0001 and log2FC > |2.5|). The first four letters of the bacterial families are indicated for each ASV. ASV numbering is illustrated according to DADA2 output, which ranks the ASVs according to relative abundance (the lower the number, the higher the abundance of the ASV). (**E and F**) Bacterial cultures of (**E**) oropharyngeal swabs and (**F**) fecal pellets after 5.5 months exposure to RA or CS. ENTE*, Enterococcaceae; ENTE**, Enterobacteriaceae.

In the oropharyngeal samples, the *Streptococcaceae* drastically increased in relative abundance after 3 and especially after 5.5 months of CS exposure (Fig. 3A). In the fecal samples, *Muribaculaceae* and *Prevotellaceae* were the most predominant families in CS group, while the *Lachnospiraceae* were the most abundant in the RA group (Fig. 3B ). Differential abundance analysis at the endpoint of CS exposure (5.5 months) identified 50 amplicon sequence variants (ASVs) belonging to 29 bacterial families with significant different fold changes (FC) (Fig. 3C) between the two groups. Most prominently, ASV3 (*Streptococcus danielieae*) was significantly increased in the oropharynx samples from CS-exposed mice. Differential abundance analysis in the fecal samples showed 35 ASVs from 10 bacterial families which were highly significantly different (Fig. 3D). Strikingly, 23 of the 35 ASVs (71.4%) belonged to the *Lachnospiraceae*. Thus, in the fecal samples, the difference between CS and RA microbiota is mainly concentrated within the taxon *Lachnospiraceae* with most ASVs being more abundant in RA feces, while few ASVs of this taxon were more abundant in the CS group. The taxa assignments for the top 250 ASVs are illustrated (Table S1).

To corroborate the sequencing data and confirm species identification, we performed semiquantitative aerobe bacterial culture at the end of CS exposure (Fig. 3E and F). In line with the above findings, *Streptococcus danieliae* was only identified in oropharyngeal samples of CS-exposed donors (CS: 8/8, 100% versus air: 0/8, 0%). In the fecal samples, *Escherichia coli* was more prevalent in the CS group (7/7; 100%) than in the air group (2/5, 40%), while *Lactobacillus murinus* was prevalent in both groups(CS 4/7 [57%], air 2/5 [40%]). Thus, 16S rRNA gene sequencing and semiquantitative aerobe bacterial culture congruently revealed distinct microbial compositions between RA and CS donor mice after 5.5 months of exposure.

### Successful transfer of disordered microbiota to GF mice

**Fig 4:**
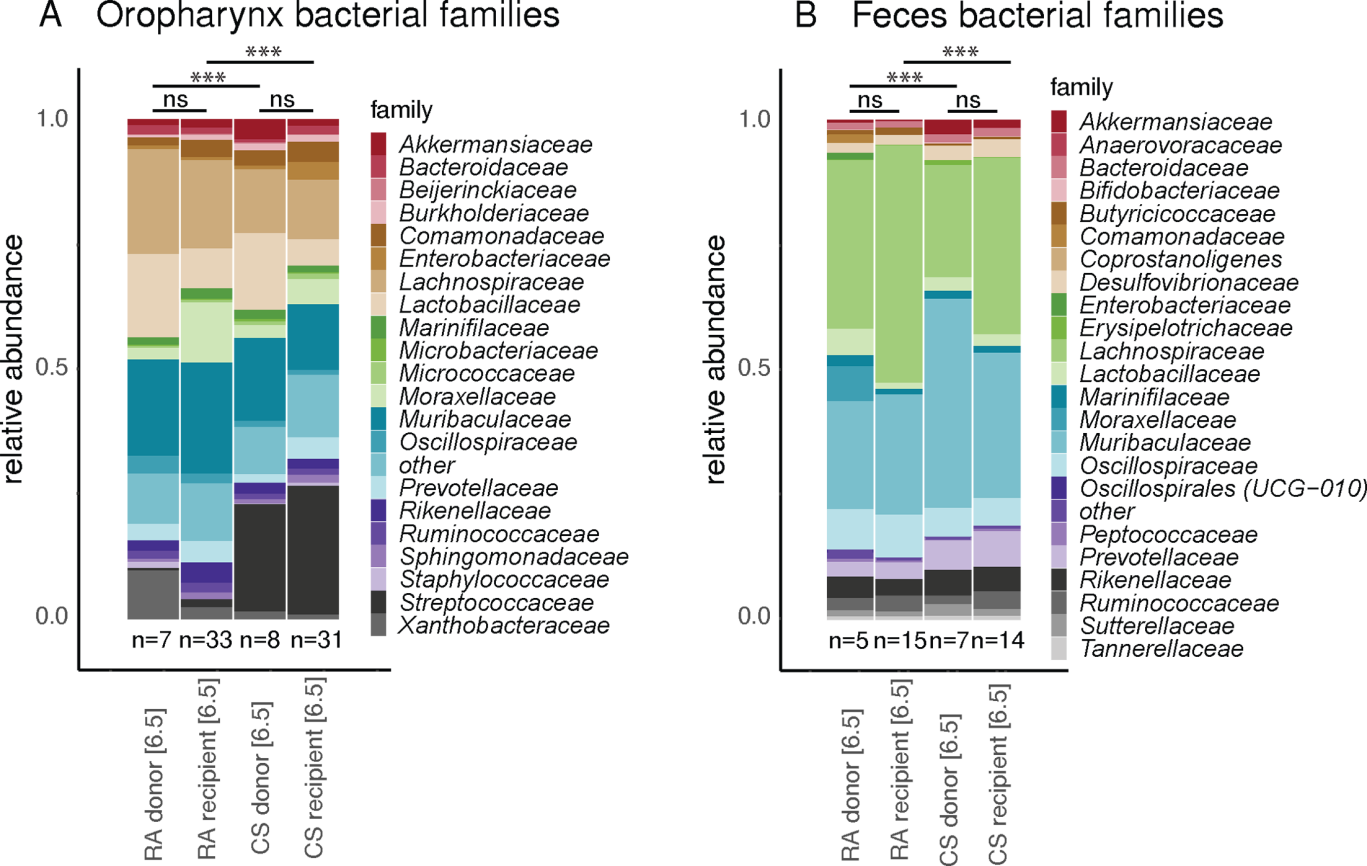
Faithful oropharyngeal and fecal microbiota transfer following cohousing of GF mice. Relative abundance of the most prevalent families in (**A**) oropharyngeal swabs and (**B**) fecal pellets of RA- and CS-exposed donor mice and their respective groups of GF recipient mice at the end of the co-housing period of 4 weeks. Data were analyzed using PERMANOVA tests (***P < 0.001).

After 5.5 months, each SOPF mouse exposed to CS or to RA was co-housed for 1 month in a cage with 3 or 4 recipient GF mice to accomplish natural microbiota transfer (Fig. 1). At month 6.5, we analyzed the fecal and oropharyngeal microbiota composition of the RA and CS-exposed SOPF donor mice to the formerly-GF recipient mice to investigate the degree of microbial transfer (Fig. 4A and B). For oropharyngeal and fecal samples, no difference in Shannon and Simpson diversity index values between donor and recipient group was observed. Similarly, analyses of the beta diversity also showed no differences between donor groups and their respective recipients. In contrast, the PERMANOVA tests showed maintained differences between the CS and the RA recipient groups (Fig. 4A and B), indicating a comprehensive and exposure-specific microbial transfer. Taken together, the data demonstrate a successful transfer of both oropharyngeal and fecal microbiota after cohousing including specific microbiota differences induced by CS exposure in formerly GF mice, which were never exposed to CS.

### CS-associated microbiota moderately increases the severity of IAV infection

**Fig 5:**
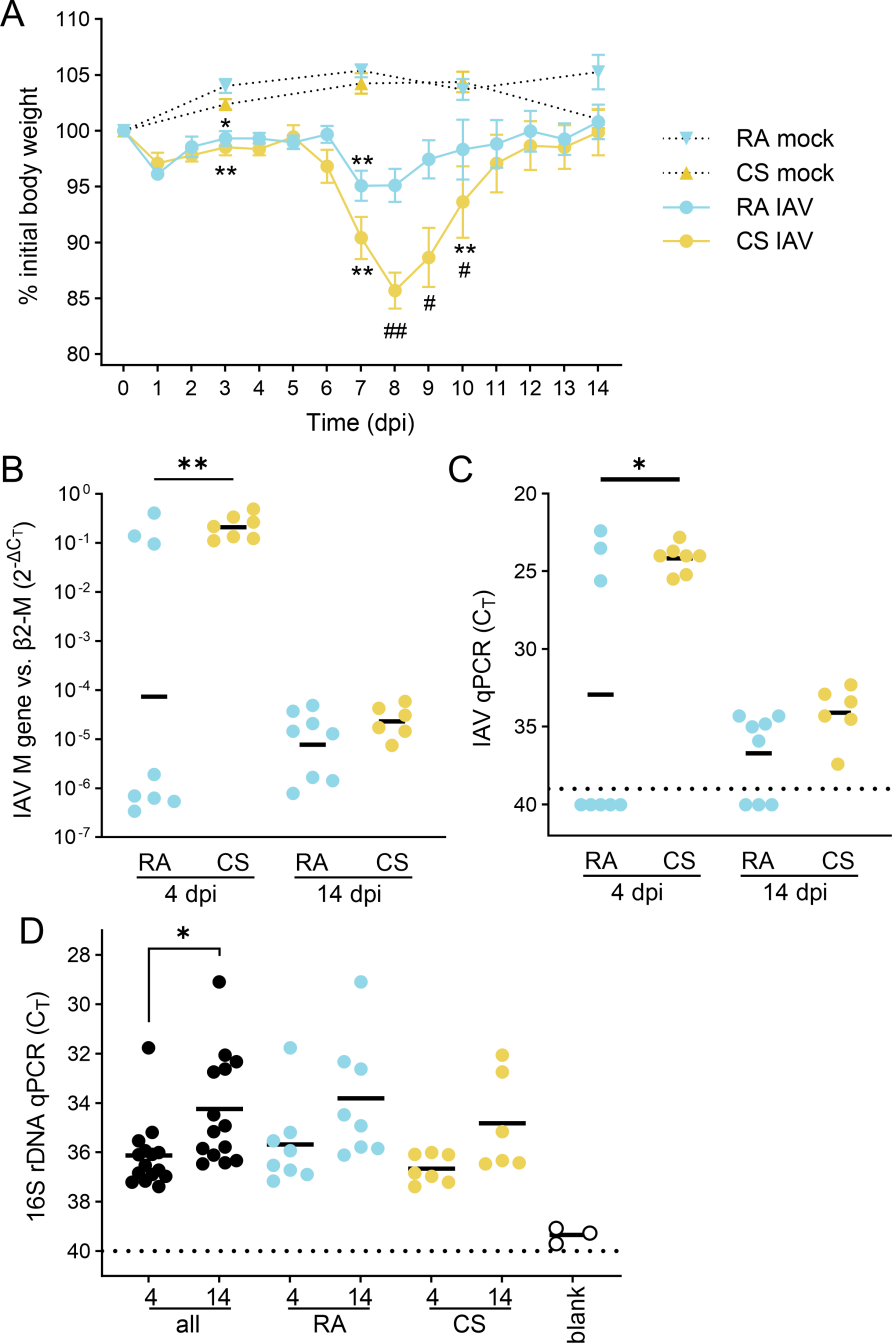
CS-associated microbiota aggravates severity of IAV infection. (**A**) Relative body weight change of colonized GF mice following 100 PFU IAV infection or control non-infected colonized GF mice. Data are shown as mean ± SD and analyzed using Wilcoxon tests (*P < 0.05, ***P < 0.001 indicate differences between infected and the respective mock-infected control; #*P* < 0.05, ##*P* < 0.01 indicate differences between CS and RA infected groups). (**B**) Virus load (IAV M gene) RT-qPCR is shown relative to the highly abundant host gene β2-microglobulin in lung homogenates of mice with RA- and CS-associated microbiota at 4 and 14 dpi. (**C**) Cycle threshold (C_T_) values for IAV M gene RT-qPCR. (**D**) Cycle threshold (CT) values for panbacter RT-qPCR of lung homogenates at 4 and 14 dpi plotted for each group and time point, or with data of both groups pooled (black). Data were analyzed by Wilcoxon test (*P < 0.05, **P < 0.01).

After successful co-housing, RA- and CS-associated microbiota recipient mice were inoculated with IAV (100 pfu/mouse). IAV infection induced body weight loss compared to mock-infected microbiota recipient groups (Fig. 5A). Moreover, the CS-associated microbiota group lost significantly more weight than the RA-associated group after 8–10 dpi. Virus load in the lungs was higher on 4 dpi compared to 14 dpi in the CS recipient group (Fig. 5B and C). No differences in viral load in the lungs were observed between the 4 dpi compared to 14 dpi in the RA recipient group. We also noted a dichotomy within RA group at 4 dpi. We hypothesize that the source of the variability may be with the infection dose. We used a relatively low inoculum (100 pfu/mouse) because we did not want to risk applying a lethal dose not knowing the actual status of the microbiota reconstitution of the GF mice. The data suggest a more rapid IAV clearance in a subgroup of RA-reconstituted mice. Interestingly, we also observed an increase of total amount of bacteria in the lung at day 14 compared to day 4 after infection. (Fig. 5D). The results of the histopathologic assessment after 4 and 14 dpi are summarized in Table 1. All IAV-infected mice showed a moderate to severe multifocal to diffuse interstitial pneumonia, primarily consisting of a lymphohistiocytic infiltrate with the presence of few neutrophils, with multifocal to diffuse expansion to peripheral lung regions. At 14 dpi, intra-alveolar inflammation was observed in a high fraction of CS-associated (4/6; 67%) and fewer air-associated (3/8; 38%) samples (and only one case at day 4 in the CS-associated group; 14%). In cases with intra-alveolar inflammation, the affected tissue areas typically showed a variable degree of hyperplasia of alveolar type 2 epithelial cells. Analysis of the lungs of the mock-infected groups revealed no structural and pathological differences between groups suggesting that microbiota transfer alone did not induce emphysematous changes associated with CS exposure.

**TABLE 1:** Histopathologic assessment of H&E-stained sections from formalin fixed paraffin embedded lung left lobes sampled on 4 and 14 dpi with IAV^*a*^

Mouse ID	dpi	RA or CS	Expansion to peripheral lung regions	Pulmonary atelectasis	Peribronchial inflammation	Perivascular inflammation	Interstitial inflammation	Intraalveolar inflammation	Bronchial epithelial necrosis	Infiltration by neutrophils	Infiltration by macrophages	Infiltration by lymphocytes	Alveolar edema	Perivascular edema	Hyperplasia of type 2 alveolar epithelial cells	Hemorrhage (interstitial/ intraalveolar)
GF14-0	4	RA	2	1	0	0	2	0	0	1	2	2	0	0	0	0
GF14-1	4	RA	2	1	0	0	2	0	0	1	2	2	0	0	0	0
GF14-2	4	RA	2	2	0	0	2	0	0	1	2	2	0	0	0	0
GF14-3	4	RA	2	1	0	0	2	0	0	1	2	2	0	0	0	0
GF14-5	4	RA	2	1	0	0	2	0	0	1	2	2	0	0	0	0
GF7-0	4	RA	2	2	0	1	2	0	1	1	2	2	0	0	0	0
GF7-1	4	RA	2	1	1	0	2	0	1	1	2	2	0	0	0	0
GF10-0	4	CS	2	1	0	0	2	0	0	1	2	2	0	0	0	0
GF10-1	4	CS	2	1	0	0	2	0	1	1	2	2	0	0	0	1
GF10-3	4	CS	2	2	0	0	2	0	0	1	2	2	0	0	0	0
GF9-0	4	CS	2	1	0	0	2	0	0	1	2	2	0	0	0	0
GF9-1	4	CS	2	2	0	0	2	0	0	1	2	2	0	0	0	0
GF9-2	4	CS	2	2	0	0	2	0	0	1	2	2	0	0	0	0
GF9-4	4	CS	2	2	0	0	2	0	0	1	2	2	0	0	0	0
GF15-0	14	RA	2	1	0	1	2	0	0	1	2	2	0	0	0	0
GF15-1	14	RA	2	2	0	0	2	0	0	1	2	2	0	0	0	0
GF15-2	14	RA	2	2	2	2	2	2	2	1	2	2	2	0	2	0
GF15-3	14	RA	2	1	0	0	2	0	0	1	2	2	0	0	0	0
GF5-0	14	RA	2	1	0	1	2	1	0	1	2	2	0	0	1	0
GF5-1	14	RA	2	1	2	2	2	2	0	1	2	2	0	0	1	0
GF5-2	14	RA	2	1	0	0	2	0	0	1	2	2	0	0	0	0
GF5-3	14	RA	2	1	0	0	2	0	0	1	2	2	0	0	0	0
GF6-0	14	CS	2	1	0	0	2	2	0	1	2	2	1	0	1	0
GF6-2	14	CS	2	2	0	0	2	0	0	1	2	2	0	0	0	0
GF6-3	14	CS	2	2	2	2	2	2	0	1	2	2	1	0	0	0
GF6-4	14	CS	2	1	0	0	2	2	0	1	2	2	0	0	0	0
GF8-1	14	CS	2	2	0	1	2	2	0	1	2	2	2	0	2	0
GF8-3	14	CS	2	1	0	1	2	2	0	1	2	2	1	0	2	0

^*a*^ Mouse ID indicates cage number and respective animal number.

### IAV infection disorders oropharyngeal microbial communities

**Fig 6:**
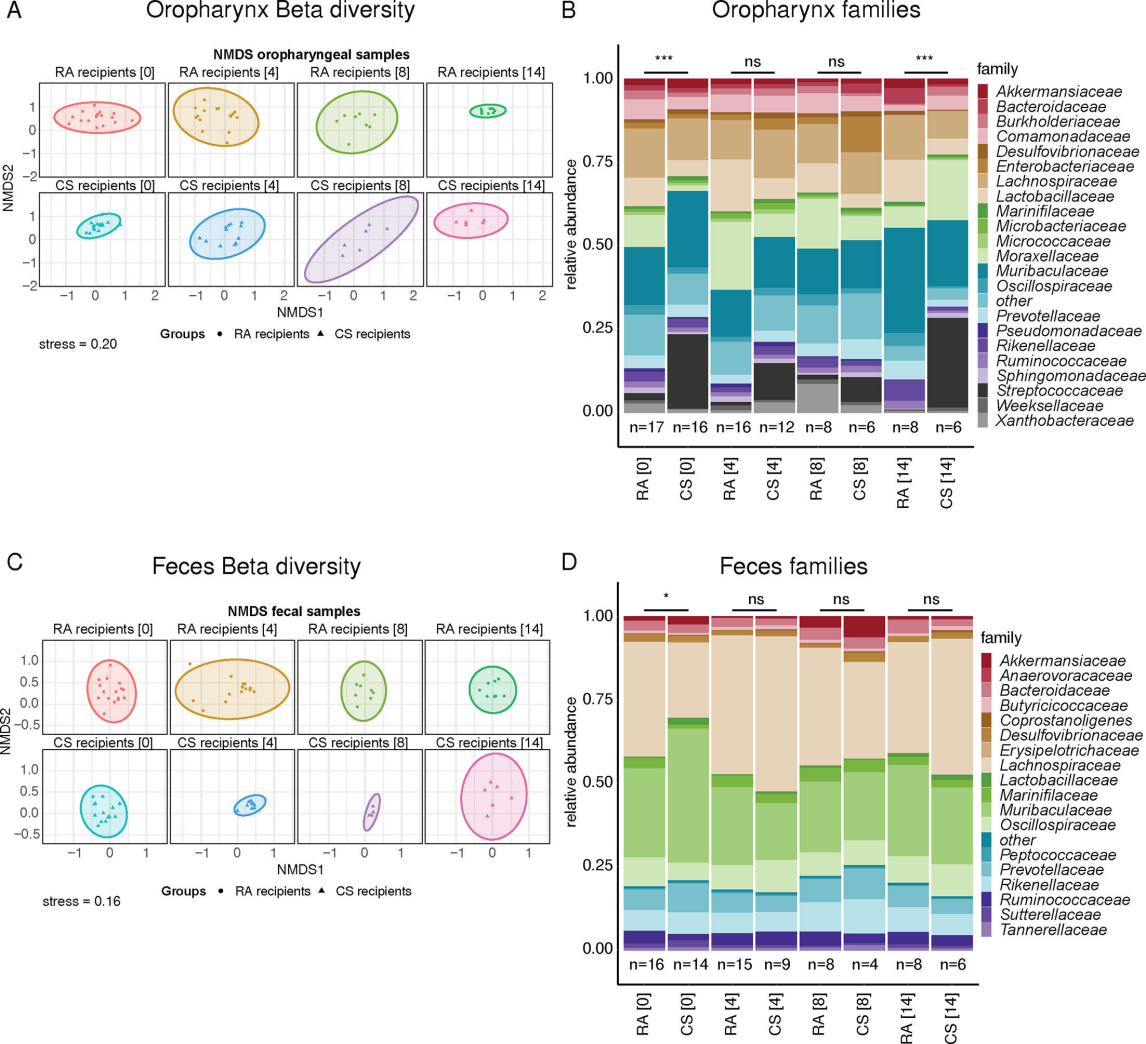
Beta-diversity and relative abundances of bacterial families during IAV infection. (**A and C**) Non-metric multidimensional scaling of Bray-Curtis distances depicted in individual panels for (**A**) oropharyngeal swabs and (**C**) fecal pellets at indicated dpi. (**B and D**) Relative abundance of the most prevalent bacterial families averaged per group in (**B**) oropharyngeal swabs and (**D**) fecal pellets at indicated dpi. Data were analyzed using PERMANOVA tests.

We next investigated the effects of IAV infection on the oropharyngeal and fecal microbiota of colonized GF mice at 0, 4, 8, and 14 dpi (Fig. 6A through D). Beta-diversity analysis showed different microbiota compositions in the oropharynx between infected CS- and RA-associated microbiota recipient mice at the time of infection (0 dpi) (*F* = 2.79, *P* = 0.001). These differences were attenuated and no longer present at the early (4 dpi) and peak (8 dpi) phases of the infection, but differences were restored after recovery at 14 dpi (*F* = 3.13, *P* = 0.001) (Fig. 6A and B). The higher abundance of Streptococacceae on 0, 4, and 14 dpi in the oropharynx of CS microbiota recipients was particularly striking and the principal cause for the divergence in the composition of the two types of airway microbiota (*P* < 0.0001; Fig. 6B). In the fecal samples, analysis of beta-diversity showed differences between the groups on 0 dpi (PERMANOVA, *F* = 2.59, *P* = 0.031), but not at the end of the experiment at 14 dpi (*F* = 0.79, *P* = 0.060; Fig. 6C and D). The differences at 0 dpi were mainly due to differences in relative abundances of Lachnospiraceae, Muribaculaceae, Oscillospiraceae, and Prevotellaceae (Fig. 6D).

In mice of the non-infected groups, the upper respiratory tract and fecal microbiota composition were evaluated at the end of the co-housing period and then weekly for 4 weeks. As expected, the oropharyngeal microbiota at week 0 corresponded to the composition of the microbiota of the respective groups of RA and CS recipient mice at 0 dpi. In particular, a higher relative abundance of *Streptococcaceae* was observed in the CS recipients compared to the RA recipients throughout the follow-up period. Some variation in the relative abundance of the main families (Lachnospiraceae, Lactobacillaceae, Moraxellaceae, Muribaculaceae) was observed in the oropharyngeal samples of RA and CS recipients. These inconsistencies likely due to the inherent variability associated with low-density samples such as oropharyngeal swabs. In contrast, the microbiota composition of the high-density fecal pellet samples remained largely unchanged for the duration of the study up to 4 weeks after the end of co-housing (Fig. S1 and S2).

In summary, IAV infection impacted the oropharyngeal and the fecal microbiota differently. Existing differences between oropharyngeal CS-associated and control microbiota were more pronounced after recovery of IAV infection. Hence, the microbiota composition of the CS and RA groups was driven further apart by IAV infection which was more pronounced in the oropharyngeal than on the fecal microbiota.

### Lung microbiota during IAV infection

**Fig 7:**
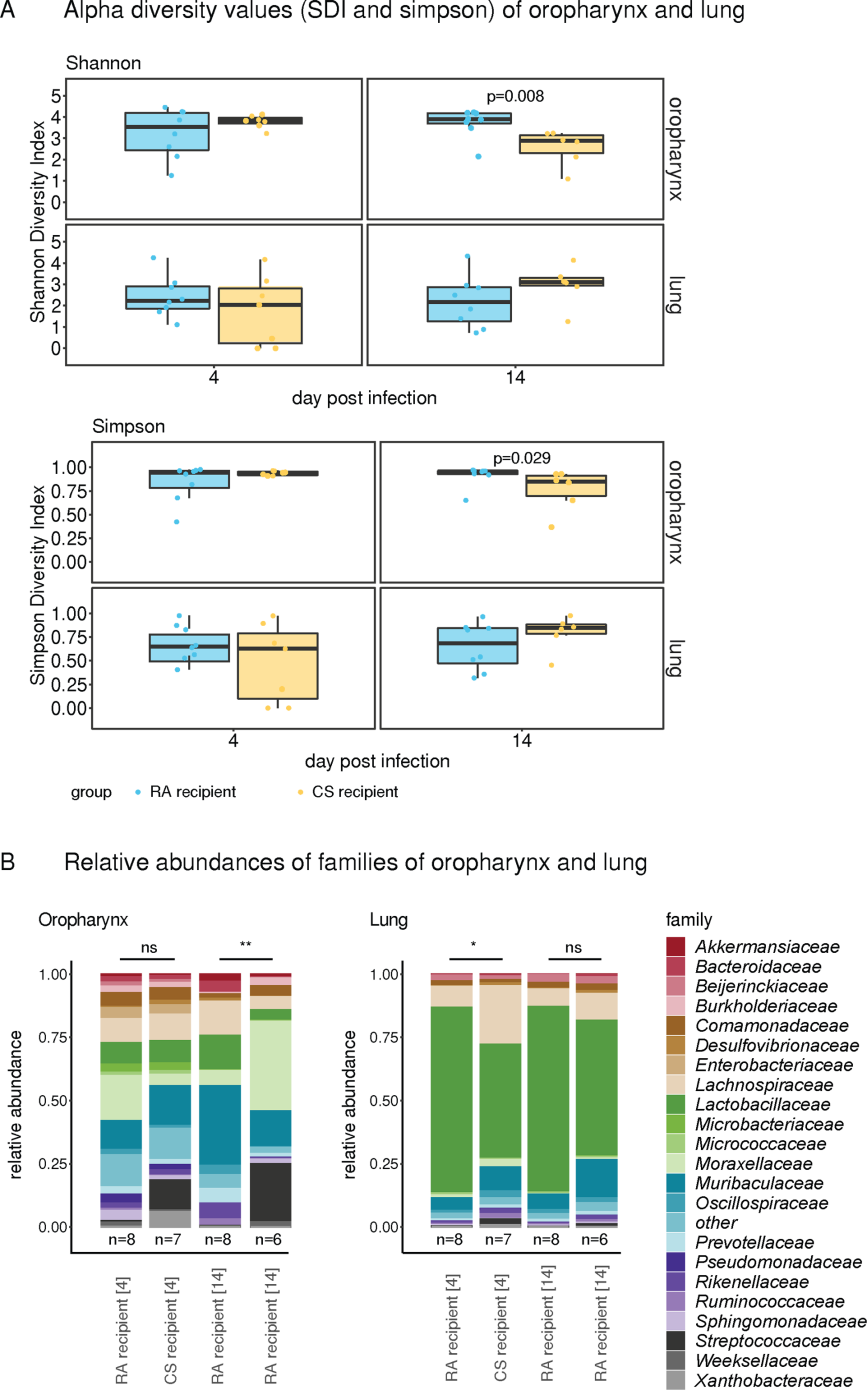
Microbial communities of upper and lower airway samples during IAV infection. (**A**) Alpha diversity values of the oropharynx and lung microbiota shown as (**A**) Shannon diversity index (SDI) and (**B**) Simpson index of colonized GF mice on 4 and 14 dpi with IAV. Data were analyzed by Wilcoxon ranked sum tests, and *P*-values are indicated. (**B**) Relative abundance at family level for oropharyngeal and lung microbiota at 4 and 14 dpi. Each bar represents the averaged community composition for the number of mice indicated below.

To investigate whether differences noted in the upper respiratory tract (URT) microbiota were also reflected in the lower respiratory tract (LRT), we juxtaposed the oropharyngeal and the lung 16S rRNA gene sequence analysis at 4 and 14 dpi for each mouse. Both diversity indices were lower in the oropharynx samples of the CS group at 14 dpi. In contrast, no differences in alpha-diversity were observed between CS- and RA-associated groups in the lungs at 4 and 14 dpi (Fig. 7A). In both groups, the LRT microbiota was less diverse than in the URT at 4 and 14 dpi and was strongly dominated by *Lactobacillaceae* (Fig. 7B). Beta-diversity of the LRT microbiota was different between the two groups at 4 dpi (*F* = 2.38, *P* = 0.03), but not at 14 dpi (*F* = 1.35, *P* = 0.2). Unfortunately, the lack of data for the LRT at 0 dpi, or from mock-infected mice at 4 and 14 dpi, limits further interpretation of the evolution of the microbiota composition in the LRT following IAV infection. In the URT, however, IAV infection appeared to have transiently erased differences in microbiota composition between the two groups during the acute phase (4 and 8 dpi) of IAV infection, but the differences between CS and RA groups reappeared on 14 dpi to the same extent as what was observed at 0 dpi (Fig. 6B and 7B left panel).

## DISCUSSION

CS has been shown to modify the microbiota but whether and to what extent it influences the severity of influenza A virus infection has remained unclear. We show that gut and oropharyngeal microbiota are altered by chronic CS exposure and that the dysbiosis is efficiently acquired by groups of GF mice cohoused with a single microbiota donor mouse previously exposed to CS. Moreover, we present evidence that CS-associated disordered microbiotas increase the severity of influenza A infection in colonized GF mice never exposed to CS.

According to the World Health Organization (WHO), tobacco is the number one cause of preventable death, with more than 8 million deaths each year worldwide (https://www.who.int/) (13). While mechanisms are unclear, CS is also associated with changes of the microbiota in humans that is generally characterized by a loss of diversity (14–16). Similarly, decreased microbial diversity was reported in the oropharynx and gut of mice chronically exposed to CS compared to age-matched controls exposed to air (6). Therefore, the disordering of the microbiota and its consequences affect not only those organs that are in direct contact with the smoke, such as the oral cavity or the airways, but may also involve distant organs, such as the gut and genitourinary tract (17). Here, we found that the Shannon alpha diversity index values of the oropharyngeal microbiota of SOPF mice declined more rapidly in the CS exposed compared to the RA-exposed group. Because they are coprophagic, GF mice successfully acquire the gut microbiota of cohoused donor mice. However, not all microbiotas are equal and autochthonous, host-adapted microbiota tend to have the upper hand when competing for an empty niche (18). When groups of GF mice were each cohoused for 1 month with a single mouse chronically exposed to CS or to RA, the GF mice strikingly harbored a microbiome that was highly congruent with the donors’ microbiota relative composition. This indicates that the cohousing approach allows the acquisition of a disordered airway and gut microbiota induced by CS in GF mice that were never exposed to the noxious smoke stimulus.

In the fecal samples, CS reduced the bacterial diversity within the *Lachnospiraceae* and increased the relative abundance of *Lactobacillaceae* compared to the control RA-exposed mice. Consistently, a recent study also found that CS exposure leads to a decrease in the relative abundance of *Lachnospiraceae* and a respective increase in *Lactobacillaceae* in the gut microbiota (19). *Lachnospiraceae* are widely accepted as core component of a healthy gut microbiota and are one of the main providers of short-chain fatty acids (SCFAs) (20, 21) which are important in immune regulation via the gut-lung axis (22). Importantly, it has been described that gut dysbiosis during influenza disease contributes to pulmonary streptococcal superinfection through alteration of SCFA production (23).

After IAV intranasal challenge, GF mice colonized with CS-associated microbiota had a relatively more severe course of infection than the RA microbiota control group based on body weight loss and microscopic lung inflammation. Thus, when the effects of chronic CS exposure are subtracted, the diverged microbiota in airways and/or gut contributes to the course of the lung viral infection. Conversely, the virus infection also transiently modified the microbiota. We identified differentially abundant bacterial taxa between the CS and RA groups. Most prominently, the abundance of *Streptococcaceae* increased in the oropharynx of the CS-exposed mice and of the corresponding recipient GF mice compared to the control groups. Higher fractions of *Streptococcaceae* in the microbiota have been associated with various oral and respiratory diseases as well as CS exposure (24–26). Moreover, influenza A disease outcomes are worsened by bacterial superinfection (27), particularly with *Streptococcus pneumoniae* and *Streptococcus pyogenes* (28). In our model, we clearly observed increased abundance of *Streptococcaceae* (specifically *Streptococcus danieliae*) in the CS-associated group, which also presented more severe disease on 8–10 dpi. Bacterial clearance and susceptibility to streptococcal influenza superinfection are associated with neutrophil and macrophage lung tissue infiltration (29). However, no difference in histopathological analysis on 4 and 14 dpi was observed between the two groups of colonized GF mice. This may be explained, in part, by the timing of the sampling that aimed to identify changes in the microbiota in the acute and recovery phase of the infection. Retrospectively, these time points did not cover the peak of body weight loss observed on 8–10 dpi, where pathological differences may have been greater. The microbial composition returned to baseline after undergoing qualitative changes in consequence of acute influenza A infection. This has been previously suggested during influenza A virus infection for the intestinal microbiota (30). In a previous study, we directly investigated changes in the oropharyngeal microbiota of mice directly exposed to cigarette smoke and following smoking cessation. We found that the changes URT microbiota induced by chronic (6 month) CS exposure was reversed 3 months after CS exposure cessation, and no significant difference was observed compared to age-matched control mice exposed to RA (6). Here, it would be speculative to assume that a similar outcome with a reversal of the URT dysbiosis would occur in the colonized GF mice. On the contrary, colonization of GF mice tends to be more permanent, unless mice are subsequently exposed to better adapted commensals, as shown previously for the gut microbiota (18).

We also studied the composition of the lung microbiota during IAV infection, which provides further insight into interactions of host pulmonary responses with the LRT microbiome (31). It had been suggested that *Lactobacillaceae* are associated with mice not previously exposed to CS (32, 33). In line with the reported findings, the LRT microbiota of our study predominantly contained *Lactobacillaceae,* and notably, the relative abundance of *Lactobacillaceae* was decreased in the CS-associated group. It has been suggested that *Lactobacillus murinus*, a bacterial species within the *Lactobacillaceae*, could potentially provide a barrier against pneumococcal colonization (34) and its reduction serves as a biomarker for IAV infection (35). Importantly, we confirmed 16S rRNA gene amplicon sequencing data by performing bacterial cultures in parallel which is of high importance when working with low-density samples which may be prone to reagent contamination (36). Finally, we observed a lower bacterial content in the lungs at the 4 dpi compared to 14 dpi, which may suggest the loss of ecological niches that may become vulnerable to bacterial invasion (37). However, since lung samples are collected at post mortem, longitudinal analysis of LRT cannot be generated for the same animals. Moreover, the lack of data regarding the bacterial content at 0 dpi does not permit a definitive statement regarding loss bacterial density at 4 dpi relative to 0 dpi. While we observed lower *Lactobacillaceae* in the lungs and higher *Streptococcaceae* in the upper respiratory tract of CS microbiota colonized GF group, we observed no colonization or proliferation of *Streptococcaceae* in the LRT upon IAV infection indicating that SOPF commensal species lack pathogenic potential to induce bacterial superinfection of the LRT. The importance of the microbiota of the LRT in the pathogenesis and severity of lung diseases remains controversial (38, 39). Emerging experimental and epidemiological evidence also highlight cross-talks between the intestinal microbiota and the lungs known as the “gut–lung axis” (22, 40). Yet, the causal relationship between CS exposure, gut and respiratory tract microbiome alterations, and viral infections needs to be further studied.

### Conclusions

Mice with CS-associated microbiota present a worse disease course of IAV infection than the control group. We, therefore, conclude that CS-induced disordering of the microbiota contributes to the severity of influenza A infection independently of the direct noxious effects of tobacco-induced cellular and structural lung damage.

## MATERIALS AND METHODS

### Study design, CS exposure, and microbiota transfer

Female C57BL/6JRj mice with a certified specific and opportunistic pathogen-free (SOPF) status (Janvier Labs, France) were obtained at the age of 4 weeks and randomized in two groups at the Department of Biomedicine, University of Basel. After one month of acclimatization, one group was exposed to CS for 5.5 months to establish a CS-associated microbiota as described before (6). The control group was kept in the same facility and exposed to HEPA-filtered room air (RA). Both groups were then transferred to the Institute of Virology and Immunology (IVI). At the same time, germ-free (GF) C57BL/6J female and male mice were also obtained from the Clean Mouse Facility, University of Bern. Groups of 4 or 5 GF mice of the same sex were randomly co-housed for 1 month with one mouse previously exposed to CS or to RA. The efficiency of microbiota transfer was verified using fecal pellet and oropharyngeal swabs of microbiome donors and colonized GF mice before and after the cohousing period (Fig. 1).

### Sample collections for microbiota characterization

Fresh fecal pellets were collected in DNA/RNA-free tubes by immobilizing the mouse in prone position and gently lifting the rear end up, to initiate defecation. Ultra minitip flocked swabs (FLOQSwab, 516CS01, Copan) were used for oropharyngeal microbiota sampling as described (6). Fecal pellets and swabs collected for sequencing were immediately immersed in 4°C lysis buffer and subsequently frozen at −20°C until DNA extraction (QIAGEN, DNeasy Blood & Tissue Kit, Cat. No. 69504).

### Bacterial culture and species identification

At the end of CS or air exposure (5.5 months), oropharyngeal swabs and fecal pellets of microbiota donor mice were placed in 500 µL of NaCl, vortexed vigorously, plated on citrated sheep blood agar (CSBA), MacConkey agar (MAC), and Columbia naladixic acid agar (CNA) plates (100 µL/plate), and incubated at 37°C in aerobic and anaerobic conditions. After 24 h and 48 h, phenotypically distinct colonies were scored semi-quantitatively for high (+++), medium (++), or low (+) growth on CSBA plates. The colonies were phenotypically sorted, chemically inactivated, and DNA isolated for 16S rRNA typing with the V1-V9 primers 27F: 5′-AGAGTTTGATCMTGGCTCAG-3′ and 1492R: 5′-TACGGYTACCTTGTTAYGACTT-3′. The amplicons were Sanger sequenced (Eurofins Genomics, Germany), contigs created with SeqMan Pro v14.0.0.86, and the species identified by nucleotide blast search (blastn tool, 3.2.2021).

### Influenza A virus infection and tissue collection

Mice were anesthetized and inoculated intranasally (20 µL) with 100 plaque-forming units (PFU) of IAV (A/Puerto Rico/8/1934 [H1N1]; ATCC VR-1469). Body weight was measured daily as a proxy for disease progression. Oropharyngeal and fecal microbiota at 0-, 4-, 8-, and 14-day post-infection (dpi) and terminal collection of organs was done at 4 and 14 dpi. The right lobes were homogenized in 2 mL PBS using M tubes and the gentleMACS Tissue Dissociator (Miltenyi), centrifuged at 200 × *g* for 2 min, and supernatants were frozen in aliquots at −70°C. Aliquots were used for RNA or DNA extraction and amplification of viral RNA and bacterial DNA, as described below. The left lung lobe was fixed in formalin for 24–72 h and processed for histopathology. Lung tissue slides were stained with hematoxylin and eosin, digitalized, and scored by a board-certified animal pathologist as previously described (41, 42).

### Viral load quantification

Total RNA from lung homogenates was extracted using the NucleoMag VET kit (Macherey Nagel, Ref: 744200) on an automated system (KingFisher Flex, ThermoFisher Scientific) and RNA purity measured on a NanoDrop 2000 (ThermoFisher Scientific). A 25 µL RT-qPCR for the viral matrix M gene was carried out on 5 µL of extracted RNA template using the AgPath-ID One Step RT-PCR Kit (Applied Biosystems, Lot: 2005254). Each sample was processed in duplicates. Amplification and detection were performed using a 7500 Real-Time PCR System (Applied Biosystems) according to manufacturer protocol: reverse transcription at 45°C for 10 min, followed by PCR at 95°C for 10 min and 45 cycles of amplification (95°C^15 s^, 58°C^30 s^, 72°C^30 s^), and extension 72°C 5 min. Viral load was quantified relative to host β2-microglobulin expression by TaqMan qPCR according to the manufacturer’s protocol (Assay mM00437762_m; ThermoFisher).

### Amplicon sequencing of the 16S rRNA gene for microbiota characterization

Swabs and fecal pellets were collected in 4°C lysis buffer (Buffer AL, Qiagen, Cat. No. 19075) and stored at −20°C. DNA isolation was done using the QIAamp DNA Mini Kit (Qiagen, Cat. No. 51306). 16S rRNA gene amplification was done with the primers 515F: 5′-GTGCCAGCMGCCGCGGTAA-3′ and 806R: 5′-GGACTACHVGGGTWTCTAAT-3′. PCR conditions were 95°C for 6 min, 35 cycles (95°C^30 s^, 59°C^30 s^, 72°C^90 s^), and final extension 72°C for 5 min. For sequencing, PCR products were cleaned with the QIAquick PCR Purification Kit (Qiagen, Cat. No.: 28106). Samples were pooled at a maximum of 96 and sequenced on an Illumina MiSeq platform with paired-end technology (2 × 250 bp, v2 reagent kit).

### qPCR for quantifying bacterial load

Real-time PCR for bacterial DNA was done as described with minor modifications (43, 44), using forward primer 16 S-F1 (5′-CGA AAG CGT GGG GAG CAA A-3′), reverse primer 16 S-R1 (5′-GTT CGT ACT CCC CAG GCG G-3′), and probe 16 S-P1 (FAM- ATT AGA TAC CCT GGT AGT CCA -BHQ1). In order to validate and calibrate the qPCR results, a standard curve was created to correlate fluorescence with known template concentration. Therefore, standards were obtained by preparing 10-fold serial dilutions of a synthesized fragment of the 16S rRNA gene (5′-CGG TGC GAA AGC GTG GGG AGC AAA CAG GAT TAG ATA CCC TGG TAG TCC ACG CCG TAA ACG ATG TCT ACT AGC TGT TCG TGG TCT TGT ACT GTG AGT AGC GCA GCT AAC GCA CTA AGT AGA CCG CCT GGG GAG TAC GAA CGC AAG-3′) in nuclease-free water. The PCR mixture contained 15 µL of 2× master mix (TaqMan Universal PCR Master Mix kit [Applied Bioscience]), 1 µL of each primer (10 µM), 1 µL of the probe (5 µM), 9.5 µL sterile water, and 2.5 µL of template DNA. The PCR conditions were 2 min at 50°C and 10 min at 95°C, followed by 45 cycles (95°C^15 s^, 60°C^60 s^).

### Bioinformatic analysis and statistical analysis

In total, this study includes 445 oropharyngeal, 416 fecal, 38 lung samples, 20 negative controls, and 1 positive control (mock community) for internal pipeline quality check. Generally, the data were processed in R version 4.1.0 under macOS Big Sur 11.5.2. Microbial profiling was done with DADA2 (v1.20.0) (45). The data set was digitally decontaminated using the “combined” method in “decontam” (v1.12.0) (46). The combined method includes probabilities from the “frequency” method (identifies contaminants by inversely varying frequency with DNA concentration) and the prevalenc*e* method (identifies contaminants by increased prevalence in negative controls). Two samples were excluded from further analysis due to “decontam” cleanup. The average read number for the fecal samples was 131,385 (range 9,295–923,119) and for the oropharyngeal samples 110,923 (range 10,479–479,398). Microbial analyses were performed with the phyloseq (v1.38.0) package unless stated otherwise. For differential abundance, the R package DESeq2 (v1.32.0) was used.

Matched group comparisons were performed with Wilcoxon ranked sum tests unless stated otherwise. High-dimensional data were adjusted by multiple testing and Bonferroni or Benjamini-Hochberg adjustment as indicated. For beta-diversity analyses, pair-wise Bray-Curtis distances between samples were calculated in phyloseq (v1.38.0) with the *dist*() function followed by non-metric multidimensional scaling (NMDS) with ordinate(object, “NMDS”, “bray”). Permutational multivariate analysis (PERMANOVA) test with 999 permutations per test was with adonis() in the vegan (v2.5.7) package. This was performed on selected groups from NMDS plots. All statistically significant results are indicated in the respective figures.

## Data Availability

All raw Illumina sequencing reads for this 16S rRNA gene amplicon sequencing project were deposited in the European Nucleotide Archive (ENA) under study accession number PRJNA1054802.

## SUPPLEMENTAL MATERIAL

Supplemental figures msystems.00790-24-s0001.pdf Fig. S1 and S2.

Table S1 msystems.00790-24-s0002.xlsx Companion to Fig. 3.

ASM does not own the copyrights to Supplemental Material that may be linked to, or accessed through, an article. The authors have granted ASM a non-exclusive, world-wide license to publish the Supplemental Material files. Please contact the corresponding author directly for reuse.

## References

[1] Muscatello DJ, Barr M, Thackway SV, Macintyre CR. 2011. Epidemiology of influenza-like illness during pandemic (H1N1) 2009, New South Wales, Australia. Emerg Infect Dis 17:1240–1247. doi:10.3201/eid1707.101173 21762578 PMC3381394

[2] Finklea JF, Sandifer SH, Smith DD. 1969. Cigarette smoking and epidemic influenza. Am J Epidemiol 90:390–399. doi:10.1093/oxfordjournals.aje.a121084 5356947

[3] Arcavi L, Benowitz NL. 2004. Cigarette Smoking and Infection. Arch Intern Med 164:2206. doi:10.1001/archinte.164.20.2206 15534156

[4] Robbins CS, Bauer CMT, Vujicic N, Gaschler GJ, Lichty BD, Brown EG, Stämpfli MR. 2006. Cigarette smoke impacts immune inflammatory responses to influenza in mice. Am J Respir Crit Care Med 174:1342–1351. doi:10.1164/rccm.200604-561OC 17023734

[5] Dye JA, Adler KB. 1994. Effects of cigarette smoke on epithelial cells of the respiratory tract. Thorax 49:825–834. doi:10.1136/thx.49.8.825 8091331 PMC475133

[6] Hilty M, Wüthrich TM, Godel A, Adelfio R, Aebi S, Burgener SS, Illgen-Wilcke B, Benarafa C. 2020. Chronic cigarette smoke exposure and pneumococcal infection induce oropharyngeal microbiota dysbiosis and contribute to long-lasting lung damage in mice. Microb Genom 6:mgen000485. doi:10.1099/mgen.0.000485 33295863 PMC8116676

[7] Ichinohe T, Pang IK, Kumamoto Y, Peaper DR, Ho JH, Murray TS, Iwasaki A. 2011. Microbiota regulates immune defense against respiratory tract influenza A virus infection. Proc Natl Acad Sci U S A 108:5354–5359. doi:10.1073/pnas.1019378108 21402903 PMC3069176

[8] Abt MC, Osborne LC, Monticelli LA, Doering TA, Alenghat T, Sonnenberg GF, Paley MA, Antenus M, Williams KL, Erikson J, Wherry EJ, Artis D. 2012. Commensal bacteria calibrate the activation threshold of innate antiviral immunity. Immunity 37:158–170. doi:10.1016/j.immuni.2012.04.011 22705104 PMC3679670

[9] Lee KH, Gordon A, Shedden K, Kuan G, Ng S, Balmaseda A, Foxman B. 2019. The respiratory microbiome and susceptibility to influenza virus infection. PLoS ONE 14:e0207898. doi:10.1371/journal.pone.0207898 30625134 PMC6326417

[10] Kaul D, Rathnasinghe R, Ferres M, Tan GS, Barrera A, Pickett BE, Methe BA, Das SR, Budnik I, Halpin RA, Wentworth D, Schmolke M, Mena I, Albrecht RA, Singh I, Nelson KE, García-Sastre A, Dupont CL, Medina RA. 2020. Microbiome disturbance and resilience dynamics of the upper respiratory tract during influenza A virus infection. Nat Commun 11:2537. doi:10.1038/s41467-020-16429-9 32439901 PMC7242466

[11] Yadava K, Pattaroni C, Sichelstiel AK, Trompette A, Gollwitzer ES, Salami O, von Garnier C, Nicod LP, Marsland BJ. 2016. Microbiota promotes chronic pulmonary inflammation by enhancing IL-17A and autoantibodies. Am J Respir Crit Care Med 193:975–987. doi:10.1164/rccm.201504-0779OC 26630356

[12] Tsang TK, Lee KH, Foxman B, Balmaseda A, Gresh L, Sanchez N, Ojeda S, Lopez R, Yang Y, Kuan G, Gordon A. 2020. Association between the respiratory microbiome and susceptibility to influenza virus infection. Clin Infect Dis 71:1195–1203. doi:10.1093/cid/ciz968 31562814 PMC7442850

[13] World Health Organization. 2024. WHO clinical treatment guideline for tobacco cessation in adults. Geneva. 38954657

[14] Antinozzi M, Giffi M, Sini N, Gallè F, Valeriani F, De Vito C, Liguori G, Romano Spica V, Cattaruzza MS. 2022. Cigarette smoking and human gut microbiota in healthy adults: a systematic review. Biomedicines 10:510. doi:10.3390/biomedicines10020510 35203720 PMC8962244

[15] Prakash A, Peters BA, Cobbs E, Beggs D, Choi H, Li H, Hayes RB, Ahn J. 2021. Tobacco smoking and the fecal microbiome in a large, multi-ethnic cohort. Cancer Epidemiol Biomarkers Prev 30:1328–1335. doi:10.1158/1055-9965.EPI-20-1417 34020999 PMC8254769

[16] Zhang W, Li J, Lu S, Han N, Miao J, Zhang T, Qiang Y, Kong Y, Wang H, Gao T, Liu Y, Li X, Peng X, Chen X, Zhao X, Che J, Zhang L, Chen X, Zhang Q, Hu M, Li Q, Kan B. 2019. Gut microbiota community characteristics and disease-related microorganism pattern in a population of healthy Chinese people. Sci Rep 9:1594. doi:10.1038/s41598-018-36318-y 30733472 PMC6367356

[17] Cicchinelli S, Rosa F, Manca F, Zanza C, Ojetti V, Covino M, Candelli M, Gasbarrini A, Franceschi F, Piccioni A. 2023. The impact of smoking on microbiota: a narrative review. Biomedicines 11:1144. doi:10.3390/biomedicines11041144 37189762 PMC10135766

[18] Seedorf H, Griffin NW, Ridaura VK, Reyes A, Cheng J, Rey FE, Smith MI, Simon GM, Scheffrahn RH, Woebken D, Spormann AM, Van Treuren W, Ursell LK, Pirrung M, Robbins-Pianka A, Cantarel BL, Lombard V, Henrissat B, Knight R, Gordon JI. 2014. Bacteria from diverse habitats colonize and compete in the mouse gut. Cell 159:253–266. doi:10.1016/j.cell.2014.09.008 25284151 PMC4194163

[19] Meng L, Xu M, Xing Y, Chen C, Jiang J, Xu X. 2022. Effects of cigarette smoke exposure on the gut microbiota and liver transcriptome in mice reveal gut-liver interactions. Int J Mol Sci 23:11008. doi:10.3390/ijms231911008 36232309 PMC9569613

[20] Vacca M, Celano G, Calabrese FM, Portincasa P, Gobbetti M, De Angelis M. 2020. The controversial role of human gut Lachnospiraceae. Microorganisms 8:573. doi:10.3390/microorganisms8040573 32326636 PMC7232163

[21] Vital M, Karch A, Pieper DH. 2017. Colonic butyrate-producing communities in humans: an overview using omics data. mSystems 2:e00130-17. doi:10.1128/mSystems.00130-17 29238752 PMC5715108

[5] Dang AT, Marsland BJ. 2019. Microbes, metabolites, and the gut–lung axis. Mucosal Immunol 12:843–850. doi:10.1038/s41385-019-0160-6 30976087

[23] Sencio V, Barthelemy A, Tavares LP, Machado MG, Soulard D, Cuinat C, Queiroz-Junior CM, Noordine M-L, Salomé-Desnoulez S, Deryuter L, Foligné B, Wahl C, Frisch B, Vieira AT, Paget C, Milligan G, Ulven T, Wolowczuk I, Faveeuw C, Le Goffic R, Thomas M, Ferreira S, Teixeira MM, Trottein F. 2020. Gut dysbiosis during influenza contributes to pulmonary pneumococcal superinfection through altered short-chain fatty acid production. Cell Rep 30:2934–2947. doi:10.1016/j.celrep.2020.02.013 32130898

[24] Jetté ME, Dill-McFarland KA, Hanshew AS, Suen G, Thibeault SL. 2016. The human laryngeal microbiome: effects of cigarette smoke and reflux. Sci Rep 6:35882. doi:10.1038/srep35882 27775059 PMC5075886

[25] Huang C, Shi G. 2019. Smoking and microbiome in oral, airway, gut and some systemic diseases. J Transl Med 17:225. doi:10.1186/s12967-019-1971-7 31307469 PMC6632217

[26] Wu J, Peters BA, Dominianni C, Zhang Y, Pei Z, Yang L, Ma Y, Purdue MP, Jacobs EJ, Gapstur SM, Li H, Alekseyenko AV, Hayes RB, Ahn J. 2016. Cigarette smoking and the oral microbiome in a large study of American adults. ISME J 10:2435–2446. doi:10.1038/ismej.2016.37 27015003 PMC5030690

[27] Rynda-Apple A, Robinson KM, Alcorn JF. 2015. Influenza and bacterial superinfection: illuminating the immunologic mechanisms of disease. Infect Immun 83:3764–3770. doi:10.1128/IAI.00298-15 26216421 PMC4567631

[28] Morens DM, Taubenberger JK, Fauci AS. 2008. Predominant role of bacterial pneumonia as a cause of death in pandemic influenza: implications for pandemic influenza preparedness. J Infect Dis 198:962–970. doi:10.1086/591708 18710327 PMC2599911

[29] McNamee LA, Harmsen AG. 2006. Both influenza-induced neutrophil dysfunction and neutrophil-independent mechanisms contribute to increased susceptibility to a secondary Streptococcus pneumoniae infection. Infect Immun 74:6707–6721. doi:10.1128/IAI.00789-06 16982840 PMC1698099

[30] Yildiz S, Mazel-Sanchez B, Bonifacio JPP, Schmolke M. 2022. A single respiratory tract infection early in life reroutes healthy microbiome development and affects adult metabolism in a preclinical animal model. NPJ Biofilms Microbiomes 8:51. doi:10.1038/s41522-022-00315-x 35780244 PMC9250495

[31] Huang YJ, Charlson ES, Collman RG, Colombini-Hatch S, Martinez FD, Senior RM. 2013. The role of the lung microbiome in health and disease. A national heart, lung, and blood institute workshop report. Am J Respir Crit Care Med 187:1382–1387. doi:10.1164/rccm.201303-0488WS 23614695 PMC5155250

[32] Zhang R, Chen L, Cao L, Li KJ, Huang Y, Luan XQ, Li G. 2018. Effects of smoking on the lower respiratory tract microbiome in mice. Respir Res 19:253. doi:10.1186/s12931-018-0959-9 30547792 PMC6295055

[33] Singh N, Vats A, Sharma A, Arora A, Kumar A. 2017. The development of lower respiratory tract microbiome in mice. Microbiome 5:61. doi:10.1186/s40168-017-0277-3 28637485 PMC5479047

[34] Yildiz S, Pereira Bonifacio Lopes JP, Bergé M, González-Ruiz V, Baud D, Kloehn J, Boal-Carvalho I, Schaeren OP, Schotsaert M, Hathaway LJ, Rudaz S, Viollier PH, Hapfelmeier S, Francois P, Schmolke M. 2020. Respiratory tissue-associated commensal bacteria offer therapeutic potential against pneumococcal colonization. Elife 9. doi:10.7554/eLife.53581 PMC7723408 33287959

[35] Chen Q, Liu M, Lin Y, Wang K, Li J, Li P, Yang L, Jia L, Zhang B, Guo H, Li P, Song H. 2023. Topography of respiratory tract and gut microbiota in mice with influenza A virus infection. Front Microbiol 14:1129690. doi:10.3389/fmicb.2023.1129690 36910185 PMC9992211

[36] Salter SJ, Cox MJ, Turek EM, Calus ST, Cookson WO, Moffatt MF, Turner P, Parkhill J, Loman NJ, Walker AW. 2014. Reagent and laboratory contamination can critically impact sequence-based microbiome analyses. BMC Biol 12:87. doi:10.1186/s12915-014-0087-z 25387460 PMC4228153

[37] Yildiz S, Mazel-Sanchez B, Kandasamy M, Manicassamy B, Schmolke M. 2018. Influenza A virus infection impacts systemic microbiota dynamics and causes quantitative enteric dysbiosis. Microbiome 6:9. doi:10.1186/s40168-017-0386-z 29321057 PMC5763955

[38] Lankelma JM, Schuijt TJ, Wiersinga WJ. 2017. Reply to letter to the editor of Gut by Dickson and Cox. Gut 66:556. doi:10.1136/gutjnl-2016-311910 27196575

[39] Dickson RP, Cox MJ. 2017. Gut microbiota and protection from pneumococcal pneumonia. Gut 66:384. doi:10.1136/gutjnl-2016-311823 PMC5083249 27037327

[40] Bowerman KL, Rehman SF, Vaughan A, Lachner N, Budden KF, Kim RY, Wood DLA, Gellatly SL, Shukla SD, Wood LG, Yang IA, Wark PA, Hugenholtz P, Hansbro PM. 2020. Disease-associated gut microbiome and metabolome changes in patients with chronic obstructive pulmonary disease. Nat Commun 11:5886. doi:10.1038/s41467-020-19701-0 33208745 PMC7676259

[41] Dietert K, Gutbier B, Wienhold SM, Reppe K, Jiang X, Yao L, Chaput C, Naujoks J, Brack M, Kupke A, Peteranderl C, Becker S, von Lachner C, Baal N, Slevogt H, Hocke AC, Witzenrath M, Opitz B, Herold S, Hackstein H, Sander LE, Suttorp N, Gruber AD. 2017. Spectrum of pathogen- and model-specific histopathologies in mouse models of acute pneumonia. PLoS One 12:e0188251. doi:10.1371/journal.pone.0188251 29155867 PMC5695780

[42] Zhou B, Thao TTN, Hoffmann D, Taddeo A, Ebert N, Labroussaa F, Pohlmann A, King J, Steiner S, Kelly JN, et al.. 2021. SARS-CoV-2 spike D614G change enhances replication and transmission. Nature New Biol 592:122–127. doi:10.1038/s41586-021-03361-1 33636719

[43] Bogaert D, Keijser B, Huse S, Rossen J, Veenhoven R, van Gils E, Bruin J, Montijn R, Bonten M, Sanders E. 2011. Variability and diversity of nasopharyngeal microbiota in children: a metagenomic analysis. PLoS One 6:e17035. doi:10.1371/journal.pone.0017035 21386965 PMC3046172

[44] Hasrat R, Kool J, de Steenhuijsen Piters WAA, Chu MLJN, Kuiling S, Groot JA, van Logchem EM, Fuentes S, Franz E, Bogaert D, Bosch T. 2021. Benchmarking laboratory processes to characterise low-biomass respiratory microbiota. Sci Rep 11:17148. doi:10.1038/s41598-021-96556-5 34433845 PMC8387476

[45] Callahan BJ, McMurdie PJ, Rosen MJ, Han AW, Johnson AJA, Holmes SP. 2016. DADA2: high-resolution sample inference from Illumina amplicon data. Nat Methods 13:581–583. doi:10.1038/nmeth.3869 27214047 PMC4927377

[46] Davis NM, Proctor DM, Holmes SP, Relman DA, Callahan BJ. 2018. Simple statistical identification and removal of contaminant sequences in marker-gene and metagenomics data. Microbiome 6:226. doi:10.1186/s40168-018-0605-2 30558668 PMC6298009

