# Supplementary material for: Cigarette smoke-induced disordered microbiota aggravates the severity of influenza A virus infection
Source: mSystems. 2024 Nov 20;9(12):e00790-24. doi: 10.1128/msystems.00790-24 (PMC11651097; doi:10.1128/msystems.00790-24)
Supplement: Supplemental figures — Fig. S1 and S2. [file msystems.00790-24-s0001.pdf]

Supplementary figures

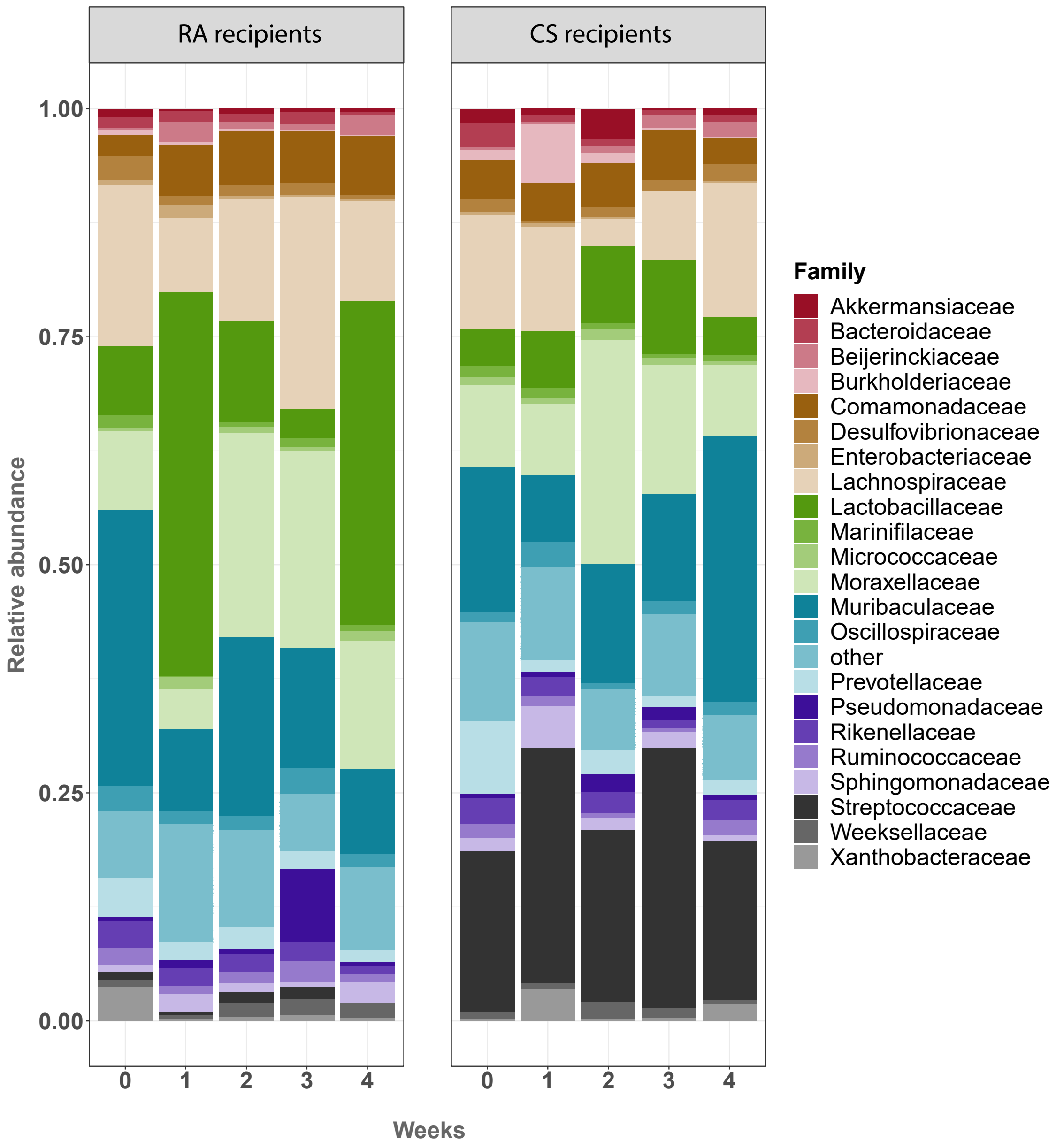

Supplementary Figure 1. Oropharyngeal microbiota analysis of recipient mice not inoculated with IAV. Relative abundance of the most prevalent bacterial families is shown for the URT of GF mice colonized with RA- and CS-associated microbiota and not infected with IAV. Oropharyngeal swabs were sampled weekly starting at the end of the co-housing period (week 0) for 4 weeks.

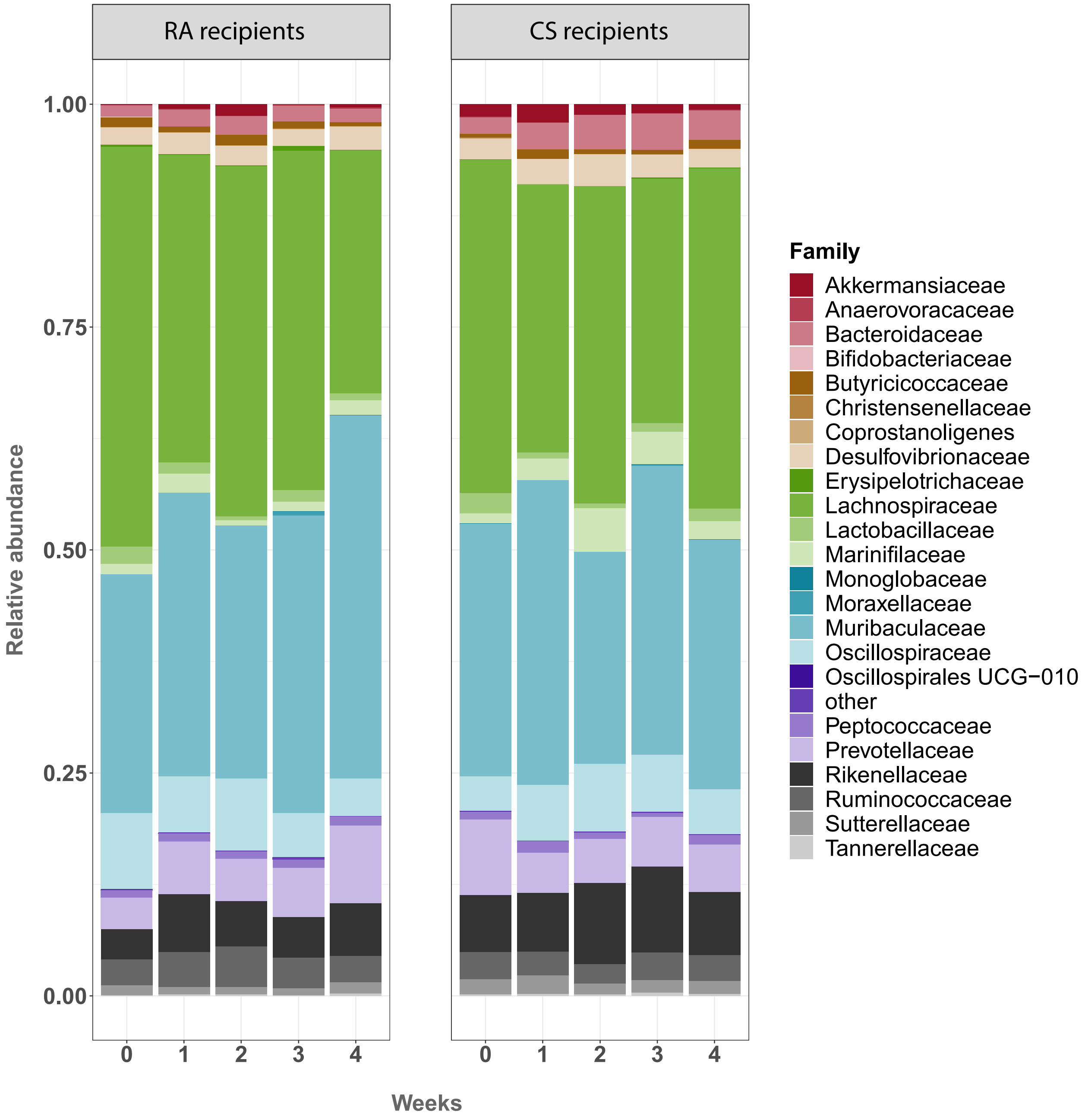

Supplementary Figure 2. Fecal microbiota analysis of recipient mice not inoculated with IAV. Relative abundance of the most prevalent bacterial families is shown for the feces of GF mice colonized with RA- and CS-associated microbiota and not infected with IAV. Fecal pellets were sampled weekly starting at the end of the co-housing period (week 0) for 4 weeks.
